# Exploring the perspectives of ‘young adults’ (18–24) who have been in formal care and their experiences of attending a socially prescribed community allotment gardening group

**DOI:** 10.1177/03080226221117447

**Published:** 2022-08-08

**Authors:** Emma J Moore, Miranda Thew

**Affiliations:** School of Health, 4467 Leeds Beckett University, Leeds, UK

**Keywords:** Community, social prescribing, young adults, care leavers, gardening, occupation-based interventions

## Abstract

**Introduction:**

Young adults who have experienced periods of time being ‘in care’ are one of the most socially deprived populations within society, with their needs largely unmet and often not fully understood. Despite the significant attempts to invest in community-based ‘social prescribing’ interventions to address such health inequalities, there is a dearth of understanding regarding how such occupation-based community groups are experienced by this particular population. This UK based qualitative study aimed to explore the experiences of young adult ‘Care Leavers’ regarding their participation in a socially prescribed community gardening group.

**Method:**

Semi-structured online interviews were conducted with six young care leavers aged between 18 and 24 years who regularly participated in a community gardening group. Interviews were recorded transcribed verbatim and analysed Braun and Clarke’s Thematic Analysis process by two researchers to maximise validity.

**Findings:**

Four key themes emerged: ‘Social belonging and connection’, ‘A safe space’, ‘Sense of achievement from active engagement’ and ‘The facilitatory aspects of nature’. The findings suggested nature-based co-occupation within a local group, enhanced social capital, self-identity and wellbeing.

**Conclusion:**

This study supports the emerging scope of using community occupation-based interventions with young adult ‘Care Leavers’ and offers an insight into their particular needs.

## Introduction

Although current health and social care service provision generally tends to focus on the needs of those who are unwell, the elderly and young children: young people transitioning to adulthood face arguably greater challenges and risks to their health and wellbeing (Wood et al., 2018). Early adulthood is a key life stage typically characterised by transitions in education, employment, financial status, housing, independent living and social peers, which without adequate support can exacerbate health and social inequalities (Marmot et al., 2020). Based on 17 key health and wellbeing indicators of young people aged 10–24 years, the United Kingdom (UK) performs less well than comparable countries in longstanding illness, the rate of 15- to 19-year-olds not in education, employment or training (NEET) and severe socio-economic deprivation (Shah et al., 2019).

A particularly vulnerable population of young adults are ‘Care Leavers’, who are defined as a person who has previously been in the formal care of statutory services for more than 13 weeks spanning their 16th birthday (Royal College of Paediatrics and Child Health, 2015). Currently, 400,000 children within England are in the social care system at any one time; this represents 3% out of a population of 12 million children (Ofsted, 2019). Nearly 40% of care leavers (aged 19 to 21) are not in any form of education, training or employment within the UK (DFE, 2019). Care Leavers, due to their chaotic and fragmented personal and family circumstances, face early transitions to independent living, typically without adequate support, and are at increased risk of social exclusion, mental illness, poverty and future long-term unemployment (NICE, 2021; DFE, 2019).

A recent national report into the inequities in health within the UK (Marmot et al., 2020) found an increase in health inequalities and decline in life expectancy over the last decade in the most deprived communities and populations despite increased national investment, arguably greater demands greater understanding of the needs of such populations. The UK Government strategies to ameliorate the deleterious effects of deprivation include investing in community-based social interventions under the umbrella term of Social Prescribing (SP), which aims to support individuals’ physical, mental health and wellbeing through individual and community empowerment (Nesta, 2020). Community gardening groups are one such intervention; they are typically sited in local allotments and provide opportunities for disadvantaged populations who normally would not have access to gardens or outdoor space, further providing an accessible occupational experience for those who are socially isolated or marginalised (Local Government Association, 2009).

## Literature review

There is considerable literature concerning young adult care leavers regarding how they are over-represented in public care services, with significantly worse emotional, psychological and behavioural problems. Additionally, they are more likely to experience difficulties in interpersonal relationships compared to those who have not been in care (Fitzpatrick et al., 2019; NICE, 2021). Oakley et al. (2018) explored the complex and multifaceted challenges faced by those who had previously experienced the care system and concluded that poorer health outcomes were related to lack of socio-economic opportunities, independence and occupational restrictions in later life. Yet, although the disadvantages are well understood, the strategies and interventions to ameliorate these are not so well established nor adequately investigated (Smith, 2017).

The link between health, wellbeing and engagement in occupation is well established in occupational therapy and science literature, with the use of occupation-based and focused interventions at the heart of obtaining improvement in health outcomes (Wilcock and Hocking, 2015). Further, it is clear that engagement in meaningful and purposeful occupation enables personal identity and thereby sense of ‘self’ (Wilcock, 2006). Albeit there is little established literature regarding how engagement in occupations within this population could potentially promote the health and wellbeing.

However, there is some evidence of how health can be benefitted from engagement the occupation of gardening, with some studies finding improvements in emotional wellbeing, social relationships, interpersonal skills and personal enrichment from a co-occupation of community gardening groups (Van Lier et al., 2017; Nicklett et al., 2016). More recently, there has been a move towards ‘green’ or nature-based interventions to enhance mental health and wellbeing (Public Health England, 2021). A focus group study by Harris (2017) which explored the influence of nature in horticulture programmes on adults with mental health conditions, found that social interaction, feelings of motivation and improved psychological and social wellbeing were the prominent benefits of participation. However, the engagement in this type of occupation was not dependant on personal interest of gardening or nature.

Bloomfield (2017) explored the impact of eight different nature-based interventions on the health and wellbeing of mental health service users (*n* = 64) undertaken as part of the ‘A Dose of Nature’ project. The community- and occupation-based groups were part of a Social Prescription service that ran across 12 weeks and focused on green space walking, agriculture and conservation. Those that completed the programme experienced a significant increase in self-reported wellbeing, social opportunities and social skills and a decrease in psychotropic medication. However, this population was not that of young adults who were previously in care.

Although the literature suggests that there is some therapeutic potential of occupations within nature and some evidence of the benefits of community gardening to health and wellbeing, there are currently no studies that understand the experiences of an occupation-based community intervention of gardening from the perspectives of young adults (18–24) who have previously been in care. Therefore, this study aimed to explore the lived experiences of a homogenous, marginalised and socially deprived population in regard to regular participation in a community- and occupation-based group.

## Method

### Research design

A qualitative methodology was chosen for this study which is integral to elucidating the subjective experiences ‘that people give to dimensions of their lives and social worlds’ (Hewitt, 2007: 1149), thereby allowing a richer understanding of the subjective experiences, emotions and views of interviewees based on Husserl’s descriptive phenomenology (Davidsen, 2014). Thematic analysis was chosen as it allows researchers to maintain a critical stance through bracketing and reflection on the understanding of the phenomena yet remains congruent to the participants voice (Chan et al., 2013).

### Recruitment

The sample consisted of six participants selected by a purposive sampling method. The inclusion criteria specified young adults aged 18–24 years who had actively engaged and regularly attended a gardening group in a community-based allotment for a minimum of 3 months. Recruitment was via a research poster which was shared on community allotments social media page within a UK socio-economically deprived area; the participant’s characteristics are outlined in Table 1.

**Table 1.** Characteristics of participants.

Participant	Age	Period of attendance (month)	Employment status	Gender
Rachel	19	4	Employed Care leaver	F
Lucy	24	3	Unemployed Care leaver	F
George	23	5	Unemployed Care leaver	M
Sarah	20	3	Unemployed Care leaver	F
Greg	24	5	Employed Care leaver	M
Ryan	22	5	Unemployed Care leaver	M

### Data collection

Semi-structured interviews were conducted and guided by an interview schedule using open-ended questions, thus enabling additional relevant themes to emanate, which is not possible with a quantitative study or a structured interview, therefore increasing the validity of the study. Each participant shared their experiences through a Skype interview, enabling participants to talk about an experience in a protective environment. Data from the interviews was digitally recorded following consent and transcribed verbatim.

### Data analysis

The process of thematic analysis began with the researchers familiarising themselves with the original data which was fully transcribed using an inductive approach and adhered to the six-step thematic analysis procedure (Braun and Clarke, 2006: 86). The principal researcher read and coded data leading to the identification of meanings, which were organised into patterns which emerged into themes and sub-themes related to the study aim and the actual context.

Firstly, analytical notes and emerging themes were documented. The researchers then re-immersed themselves into the data after each analysis to identify final refinements of the themes and to ‘identify the “essence” of what each theme is about’ (Braun and Clarke, 2006: 92). Participant’s words were used verbatim to illustrate the themes and to increase transparency and veracity of the lived experience accounts of participants.

### Ethical considerations

Ethical approval was obtained through the university local research ethics process. Written informed consent was obtained from all participants as part of this study. Although the study was identified as low risk to participants, it was imperative to minimise risk of stress from recall of any past events by implementing strategies to ensure the psychological wellbeing of the participants. A gatekeeper allowed access to the research participants and offered a point of support, as well as provision of support leaflets, providing contact numbers to available services that could be accessed after the interviews.

### Trustworthiness

Credibility of the data was achieved through transparency during the process of data analysis, with both researchers checking preliminary findings and interpretations against the raw data. The verbatim quotes of the participants’ voice illustrate the findings of this study. Furthermore, the lead researcher bracketed own thoughts and experiences via reflective notes taken both immediately after the interview and following discussions with the second researcher (Creswell, 2014).

## Findings and discussion

All participants spoke enthusiastically about their shared experience of attending the ‘Allotment Group’ which was set up as a social enterprise (SE) to promote social inclusion and health and wellbeing amongst the local community. All participants had been attending the group for at least 3 months and usually attended twice a week. The group provides a collective opportunity for members to engage in a wide range of gardening occupations including but not limited to digging, sowing crops and growing fruit and veg. Alternatively, the group provides opportunity to build raised beds and furniture for communal areas, using a wide range of materials, for example, wooden pallets that are donated to the group and opportunity for members to participate in communal cooking using home-grown produce. The majority of participants stated they lived locally to the community allotment or used public transport to access the community allotment. The demographics of the participants are described below (Table 1); pseudonyms are used in place of the actual names to preserve anonymity.

In order to illustrate the themes (see Table 2) that emerged from the analysis of the data, each theme in turn will be explored and synthesised using verbatim quotes for transparency alongside relevant literature to consider the impact of the new knowledge that this study arguably presents.

**Table 2.** Table of themes.

Key themes	Sub-themes
Social belonging and connection	Shared/Co-occupation Inclusive environment Belonging to the local community
A safe space	Safe from judgement and alienation Escapism
Sense of achievement from active engagement	Developed skills Being productive Physical and mental wellbeing
The facilitatory aspects of nature	Connection to nature Mastery of their environment

All participants described feeling a sense of social connection and belonging, not just within the group itself but also with their local community, with participants identifying that it was one of the most important motivators for engaging in a community allotment in the first place. The resultant relationships and friendships were meaningful and were sustained as described by Lucy:

*It has helped me build relationships outside of the group. I now have friends which I spend time with outside of the group.*

Further, participation in the community group appeared to be also influenced by shared engagement in an occupation where social interactions were developed through the sharing of physical tasks, and teamwork as epitomised by ‘George’:

*I helped making raised beds, that wasn’t hard physically, but I have never done it before. I had to learn how to make it from scratch with someone helping me. It was good though, because I got to … make something that everyone could see.*

This is a considerably relevant factor for care leavers as they had previously been socially disconnected due to a lack of a stable family unit (Oakley et al., 2018). This social environment provided a basis for the group to collectively respond to any challenges they faced, giving rise to feelings of self-confidence, cohesion and self-efficacy which reflects the view that individual self-efficacy and self-confidence can increase through group participation (Christiansen and Townsend, 2004).

This co-occupation of ‘doing’ (Wilcock, 2006) echoes findings from a previous study with adults experiencing mental health conditions (Bloomfield, 2017). Indeed, it was the co-occupation alongside similar ages and circumstances that the majority of participants within the study identified as factors for maintaining occupational engagement and motivation in their general activity levels. This was also a feature in research by Whatley et al. (2015) conducted with people recovering from mental ill-health, in that occupational participation when enabled by socially inclusive environments, enhanced mood and wellbeing.

Participants felt the allotment was a ‘safe space’ (Sarah) which enabled respite from the reality of living in a world where there had previously been experiences of chaos, mistrust and stress, which is a typical manifestation of previously being in care (NICE, 2021), as expressed by Greg:

*As a young care leaver, we don’t get support with community activities……We don’t get told where the support is, and on the day I got my flat, I got dropped by my social worker with no support.*

Sarah and all other participants expressed how the feeling of ‘safety’ within the environment enabled participants to express themselves and to share experiences without being scrutinised, or over analysed or made to feel alienated from the local community. Further, feeling safe was identified as an important aspect of maintaining engagement in the locally based gardening group.

*It was a safe space where I could talk about any concerns to my friends and other members of the group. Rather than having a really serious conversation, you could chat whilst you were doing something, making it light-hearted and it felt like a lot better environment to be able to talk about things. (Sarah)*

Indeed, the shared occupation within a local, accessible, community allotment environment reflects the sense of restoring ‘social justice’ (Hocking, 2017) which is concerned with equal access to wealth, opportunities and privileges within a society. The participants’ narratives were littered with phrases regarding the impact of the group environment such as ‘it’s a calming place’ and ‘holistic environment’ as expressed by Rachel:

*It felt like you were miles away from anywhere. Honestly, it was really peaceful and secluded away from the stresses of life! [Pause], I would describe it as being a holistic environment. It made me feel calm and happy, I was able to focus on what I was doing.*

A sense of belonging and ‘inclusion’ were embedded throughout most of the themes within this study, which had a positive impact on occupational engagement and facilitated positive emotional responses, sharing an occupation with others from a similar childhood background seemingly countered the feelings of abandonment and alienation from their local community, and instead fostered feelings of acceptance and trust as Sarah acknowledged:

*It felt really nice to feel included by people you don’t know.*

*Now a days you can walk past people and smile, and they don’t acknowledge you, even so much as look at you. Whereas at the allotment you get to have a chat with people, it’s nice, because in a lot of places I don’t feel included.*

The participants experiences reflected Wilcock’s (2006) theoretical elements of occupational engagement; ergo, ‘doing’ of an occupation (gardening) ‘being’ a gardener ‘belonging’ to an inclusive gardening group and ‘becoming’ a more active member of their local community, which in turn, was an essential part of developing valued roles, identity and occupational potential which society can prejudicially deny (Hyde et al., 2017).

All participants described feelings of improved mental wellbeing, partly as a result of the environment as described earlier, but also, the positive feelings were related to the immersion in meaningful activity which was described as a healthy distraction to daily life stressors as expressed by Greg:

I tend to overthink things a lot. Being part of the group distracted me from thinking as I was constantly doing things. Because I didn’t overthink things after being at the allotment when I come to think about them, I wasn’t confusing myself. I was thinking clearer and finding solutions that I didn’t see before.

Engagement in the allotment also served as a valuable experience which emphasised the improvement in physical stamina and strength, although some stated that the demands of tending to the land led to some dry humour about the ambiguous relationship with the more strenuous aspects of the gardening, as Greg pithily observes:

I got delegated the job of digging and emptying the thing we called the pond [laughs]. It wasn’t actually a pond it was a big hole filled with water which was physically demanding!

These positive influences from the participation in the gardening group in terms of enhanced mood, self-esteem, confidence and reduced anxiety levels echo a similar study (York and Wiseman, 2012) which sought to explore people’s experiences of gardening. Ultimately, the gardening group appeared to offer a sense of freedom to escape from stress, prejudice and mentally restorative effects within a nature-based space lends itself to a recovery approach.

Another important aspect of the experience and participation in the shared occupation of ‘community gardening’ was that it enabled them to learn new skills or improve existing skills which supported them in employability, instilling feelings of self-efficacy, and increased confidence as Greg described:

*I was able to develop my horticultural skills and learn social skills that I could transfer into life and work- they can even help with getting a job [pause]…… It helped me with teambuilding and leadership skills. As a care leaver [erm], we don’t get support, so this has been a real good experience for me!*

This concept of self-efficacy, co-production and achievement were prominent concepts within the participants’ experiences; the sense of enjoyment in purposeful roles was key to the participants who previously felt they had not had a sense of achievement, as described by Ryan. *“I really enjoyed planting the veg and watching it grow, it made you feel that you had achieved something, and I looked forward to going every week.”*

All participants associated feelings of pleasure and even joy that was facilitated by being outdoors and surrounded by nature whilst engaging in the gardening, as Lucy enthusiastically stated:

*I enjoyed planting and watching food grow whilst listening to the birds and being surrounded by greenery and flowers [pause] and then eating food that we had grown- I felt proud! We would walk around picking the fruit from trees [laughs] and talk about stuff over lunch using the patio and furniture that we made. It was so much fun!*

This immersion in a meaningful occupation in a nature-based environment appeared particularly relevant to the young adults from cared for backgrounds, in that they expressed a desire to escape from past and present problematic and anxiety provoking life circumstances as epitomised by Greg:

*When I am outside, I feel my mind is clearer, [erm] I feel happier it was a world that I could escape to. [Erm] I think I would describe the allotment as a peaceful, calming and relaxing place to be.*

This relationship between health, natural outdoor spaces and wellbeing is of emerging interest across the UK (Bragg and Leck, 2017) and echoes the work of occupational therapists to enable people to carry out authentic occupations that enhance wellbeing within natural environments (Kielhofner, 2008).

Ultimately, the findings within this study illustrate how people who identify as a socio-deprived/marginalised population group can find a sense of self-identity, competency, improved mental wellbeing and mastery through co-occupation in a natural environment (Kielhofner, 2008). This supports the aim of the social prescribing agenda to use occupation to facilitate engagement in socially derived but health-related activities (Thew et al 2017).

## Strengths and limitations

This study addressed important limitations of previous similar horticultural research studies in that there is now a homogenous study of the experiences of young adult care leavers (18–24) and their participation in an occupation-based group, adding value to the knowledge of this under-represented population (NICE, 2021).

Reflexivity within the analysis of data offered a strategy for themes and coding to be checked against raw data to ensure findings are consistent with shared experiences; however, member checking could have added credibility and validity (Bryman, 2012). The homogeneity of the group members appeared to enhance the co-occupational engagement element of gardening, but it was less clear whether it was this factor that enhanced wellbeing and social connectedness.

## Implications for future research and development

This study provides the first such example of engagement in occupation-based group intervention which can enhance a sense of identity and connectedness for young adults who have previously been in care under the social prescribing agenda. As such, it provides an example of the broadening and emerging scope of occupational therapy when working with disadvantaged and marginalised populations who have unmet needs (NHS, 2019).

A future research opportunity would be perhaps the use of an ethnographic study to offer a deeper understanding of experiences and comprehensive accounts of different social phenomena to illuminate further whether it was the nature based on the occupation or the co-occupation of a homogenous group that was so relevant for this population (Huff et al., 2022).

The study did not specifically measure the effects of a socially prescribed intervention on mood, employability and community inclusivity. Therefore, studies are required that measure the outcomes and impact that outdoor community-based occupational groups can have on the health and wellbeing of young adult care leavers.

## Conclusion

This research illuminates the lived experiences of young adult care leavers and their shared sense of occupational and social injustice caused by the upheaval of not having a stable or safe home environment. This study represents the first to explore the experiences of young adults who have previously been in statutory care services and their perspectives of engaging in a community allotment/gardening group.

The findings suggest that wellbeing and identity can be disrupted by childhood and adolescent traumatic life events such as family breakdown, migration and lack of support systems. The emergent themes illustrate a sense of shared meaning of co-occupation in nature, which in turn, offered a sense of community connectedness and enhanced wellbeing; this is integral to the profession of occupational therapy and offers a tangible illustration of the value of meaningful occupation.

The findings offer an opportunity to transfer such an intervention to a wider range of disadvantaged populations, such as adolescents experiencing care, youth offenders and educational settings using an occupational justice approach, and certainly support the role of occupational therapy as part of the social prescribing agenda.

## Key findings

• ‘Community gardening’ has therapeutic value.
• Co-occupation with others from similar backgrounds fostered feelings of acceptance which countered feelings of alienation.
• Nature-based co-occupations can enhance social connectedness and inclusivity.

## What the study has added

This study provides evidence that ‘community gardening’ has therapeutic value and can serve as a therapeutic tool in contributing to advancements in occupational justice for disadvantaged population groups.
